# Diagnostic Evaluation of Pancreatic Carcinoma and Chronic Pancreatitis by Pancreatoscopy

**DOI:** 10.1155/DTE.3.177

**Published:** 1997

**Authors:** Hisao Tajiri

**Affiliations:** Endoscopy Division National Cancer Center Hospital East 5-1, Kashiwanoha 6-chome Kashiwa Chiba 277 Japan

## Abstract

We succeeded in viewing the image of pancreatic duct on a TV monitor as a sequential electronic endoscope image by connecting a converter with a charge-coupled device to an ultra-thin pancreatoscope. Spacial image processing by adaptive enhancement, using an electronic endoscope, was studied in the pancreatoscope images of 18 cases (10 with pancreatic cancer and 8 with chronic pancreatitis). As a result, it became clear that the images obtained in Peak 2 of adaptive enhancement are much better than the original images. There was an excellent effect of clearly detecting the characteristic mucosal patterns in pancreatic cancer and chronic pancreatitis. We are convinced that this method would be very useful in improving the diagnostic ability of pancreatic cancer using an ultra-thin pancreatoscope.


*Diagnostic and Therapeutic Endoscopy*, 1997, Vol. 3, pp. 177–182
Reprints available directly from the publisher
Photocopying permitted by license only

© 1997 OPA (Overseas Publishers Association)
Amsterdam B.V. Published in The Netherlands
by Harwood Academic Publishers
Printed in Singapore

### Diagnostic Evaluation of Pancreatic Carcinoma and Chronic Pancreatitis by Pancreatoscopy

HISAO TAJIRI

*Endoscopy Division, National Cancer Center Hospital East, 5-1, Kashiwanoha 6-chome, Kashiwa, Chiba 277, Japan*

*(Received 28 November 1995; in final form 20 September 1996)*

**We succeeded in viewing the image of pancreatic duct on a TV monitor as a sequential electronic endoscope image by connecting a converter with a charge-coupled device to an ultra-thin pancreatoscope. Spacial image processing by adaptive enhancement, using an electronic endoscope, was studied in the pancreatoscope images of 18 cases (10 with pancreatic cancer and 8 with chronic pancreatitis). As a result, it became clear that the images obtained in Peak 2 of adaptive enhancement are much better than the original images. There was an excellent effect of clearly detecting the characteristic mucosal patterns in pancreatic cancer and chronic pancreatitis. We are convinced that this method would be very useful in improving the diagnostic ability of pancreatic cancer using an ultra-thin pancreatoscope.**

*Keywords:* Pancreatoscope, pancreatic cancer, spacial image processing

### INTRODUCTION

We clinically tried endoscopy of the main pancreatic duct in various pancreatic diseases by inserting an ultra-thin pancreatoscope through a cannula into the normal papilla of Vater[1]. There have been few sufficient diagnostic values for an ultra-thin pancreatoscope containing only 3000 optical image fibers and the small-sized images when compared with the conventional fiberscope used for the digestive system. We first succeeded in viewing the image of the pancreatic duct on a TV monitor as a sequential electronic endoscope image by connecting a converter with a high-grade charge-coupled device to an eyepiece of an ultra-thin pancreatoscope[2,3]. We then studied spacial image processing by adaptive enhancement, using an electronic endoscope, in the pancreatoscope images of cases with pancreatic cancer and chronic pancreatitis.

Corresponding Author: Hisao Tajiri, M.D., Endoscopy Division, National Cancer Center Hospital East, 5-1, Kashiwanoha 6-chome, Kashiwa, Chiba 277, Japan Tel: 0471-33-1111 Fax: 0471-31-4724

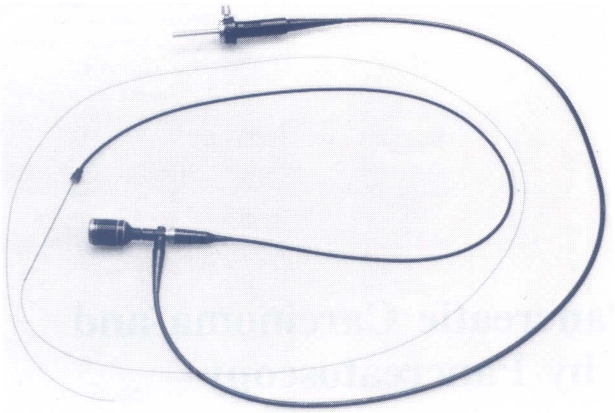

FIGURE 1A Complete view of the 0.8mm diameter pancreatoscope.

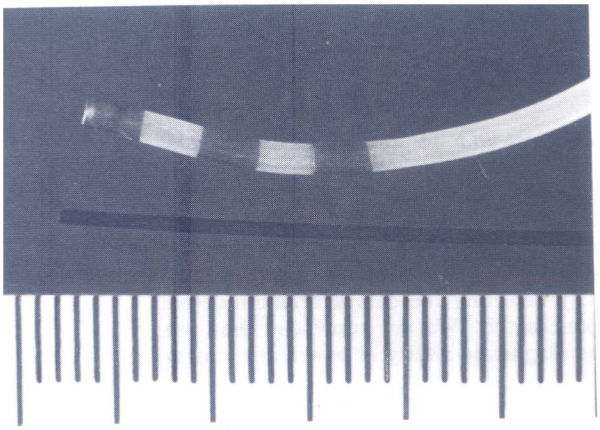

FIGURE 1B The tip of the ultra-thin pancreatoscope. A cannula shown above the pancreatoscope is 5F with a diameter of 1.7mm.

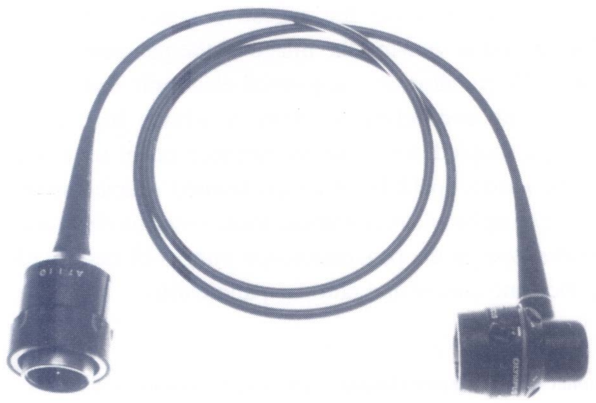

FIGURE 2 The special video converter.

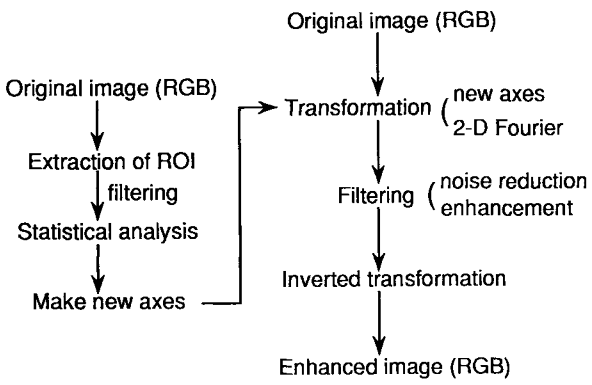

FIGURE 3 Method of the adaptive enhancement processing system.

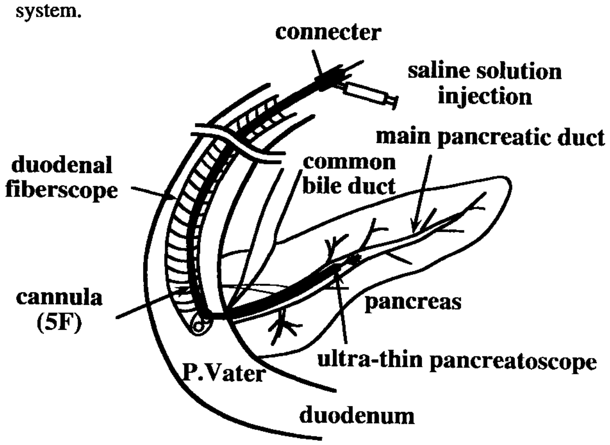

FIGURE 4 Schematic diagram of method of the ultra-thin pancreatoscope.

### MATERIALS AND METHODS

An ultra-thin pancreatoscope with a 0.8mm external diameter, a sequential electronic endoscope (Olympus Co., Ltd., EVIS 200 system), special video converter (OVC 200), U-matic VTR (Sony Co., Ltd.), and image input processing system (Olympus IP system) were employed (Fig. 1A, 1B and 2). On the IP system, analog signals from the electronic endoscope are converted to digital signals, and then processed by personal computer (Fig. 3). The adaptive enhancement processing system (according to the statistical method) makes it possible to obtain a clearer image of the fine structure pattern of the mucosa[4]. Three peak frequencies were amplified to a maximum of twice,

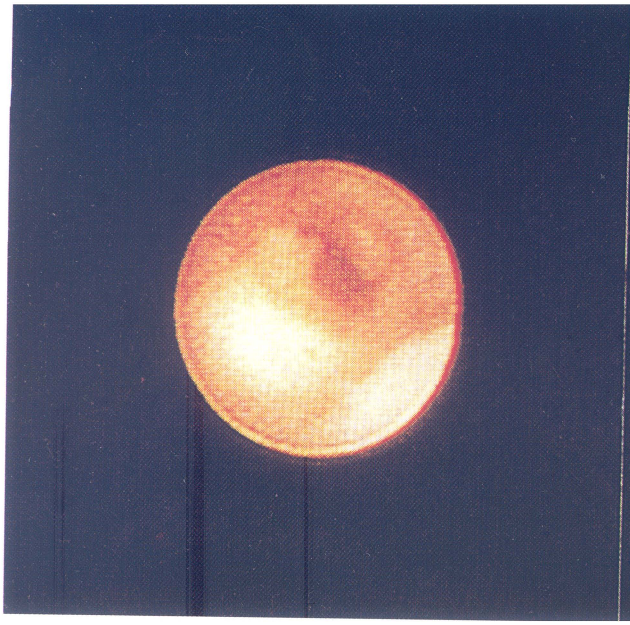

FIGURE 5A Original image of the main pancreatic duct in a case with carcinoma of the head of the pancreas.

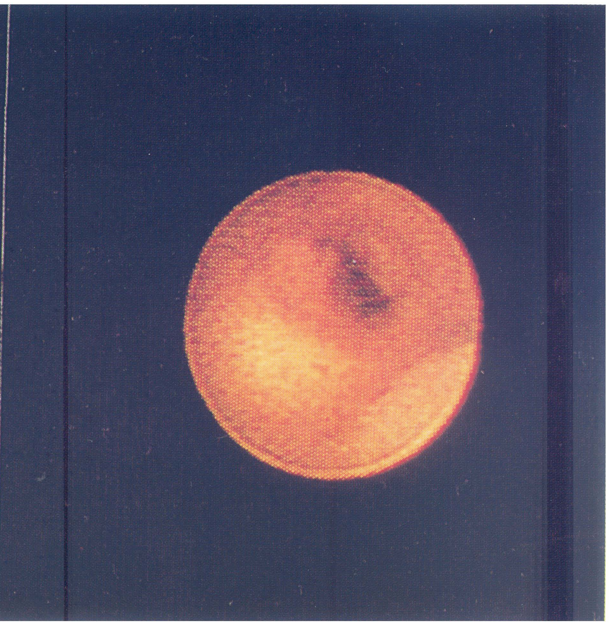

FIGURE 5B The enhanced image obtained in Peak 2 adaptive enhancement. Irregularly uneven mucosal margin due to cancer infiltration is clearly demonstrated.

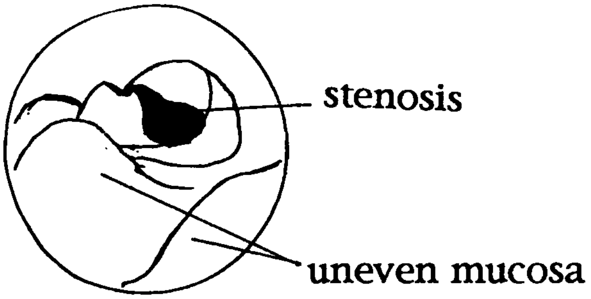

FIGURE 5C Scheme of the endoscopic picture.

3 times and 5 times respectively (Peak 2: twice, Peak 3: 3 times, Peak 5: 5 times).

A duodenal fiberscope (JF 200, Olympus Co., Ltd.) was introduced into the second portion of the duodenum, and conventional pancreatography through a cannula (5F, 1.7mm) was performed. The cannula was further advanced under fluoroscopy into the tail of the pancreas. The ultra-thin pancreatoscope was then inserted into the cannula. When several millimeters of the tip of this scope emerged from the cannula, the lumen of the pancreatic duct was observed while saline was infused into the duct through the cannula (Fig. 4).

The pancreatic duct in pancreatic cancer and chronic pancreatitis was displayed, due to each of the 3 grades of enhancement processing, on the 14-inch TV monitor. The sharpness of the construction of the lesions, peripheral color and image quality were analyzed to compare with original images by three endoscopists.

### PATIENTS

Eighteen cases (10 with pancreatic cancer and 8 with chronic pancreatitis) which undertook endoscopic examination by the ultra-thin pancreatoscope and the previous image processing from September 1992 to February 1995 were studied. Seven of the 10 patients with pancreatic cancer had carcinoma of the head of the pancreas, and the other 3 had carcinoma of the body or the tail of the pancreas. All patients agreed to participate in our study and signed an informed consent.

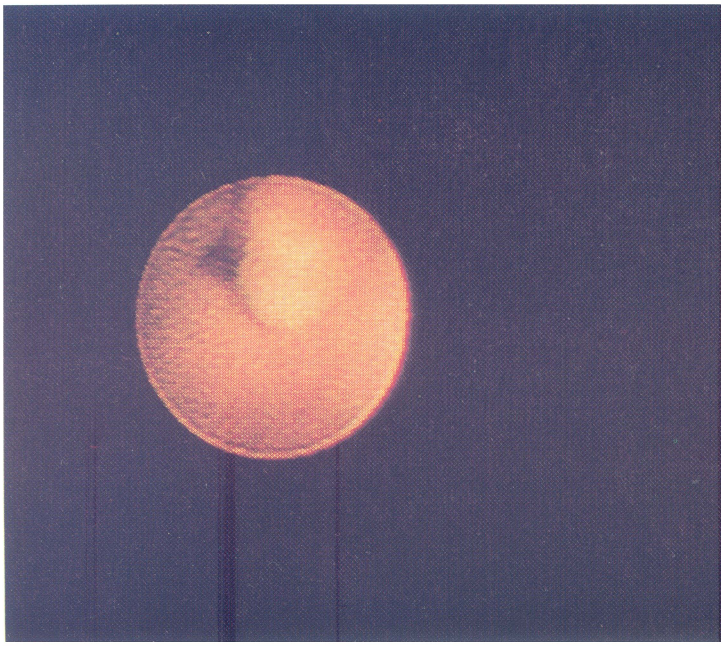

FIGURE 6A An original image of the main pancreatic duct in a case with carcinoma of the body of the pancreas.

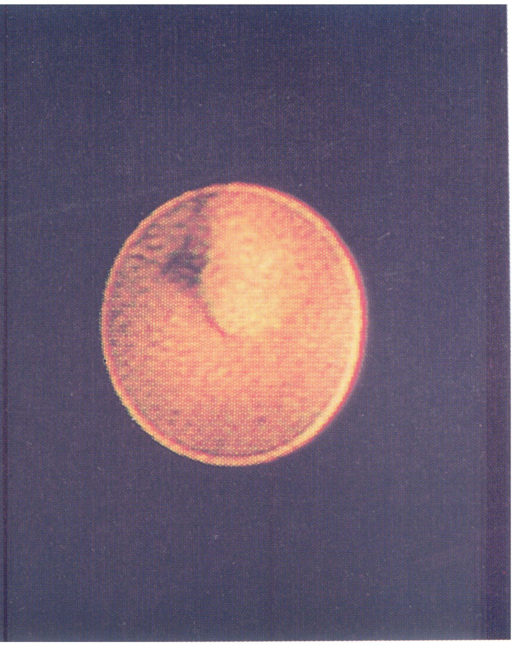

FIGURE 6B The enhanced image obtained in Peak 3 adaptive enhancement. The irregularly elevated lesion shows sharper margin.

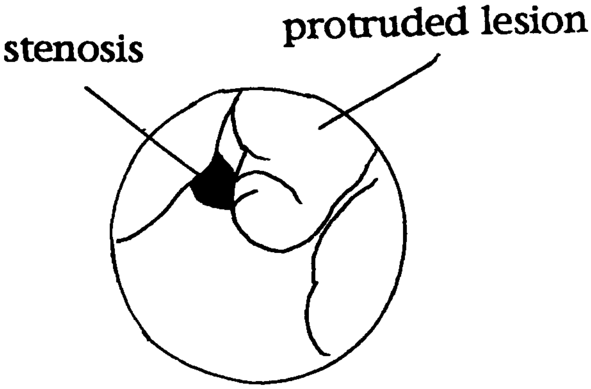

FIGURE 6C Scheme of the endoscopic picture.

### RESULTS

Images of irregular elevated lesions and friable mucosa with erosion were displayed in 8 of the 10 cases with pancreatic cancer. Extraductal compression with stenosis was disclosed in the other 2 patients. The former 8 cases, because of Peak 2 enhancement, demonstrated clearly uneven mucosa by malignant cell infiltration and peripheral color of the lesions (Fig. 5A, 5B and 5C, Fig. 6A, 6B and 6C).

A scar formation with smooth mucosa at stenotic areas was observed in 6 of the 8 cases with chronic pancreatitis. These 6 cases showed a marked enhancement effect of the fine structure of the duct (Fig. 7A, 7B and 7C). Additionally, protein plugs were displayed in 3 of the 8 cases with chronic pancreatitis. Half of all cases showed a favorable enhancement effect due to Peak 3 enhancement. Generally, image quality tended to be lower with a higher enhancement. This was the worst in Peak 5 (Table I).

### DISCUSSION

We have been advancing a new endoscopic diagnosis for the pancreatic duct system since we reported the clinical application using an ultra-thin pancreatoscope

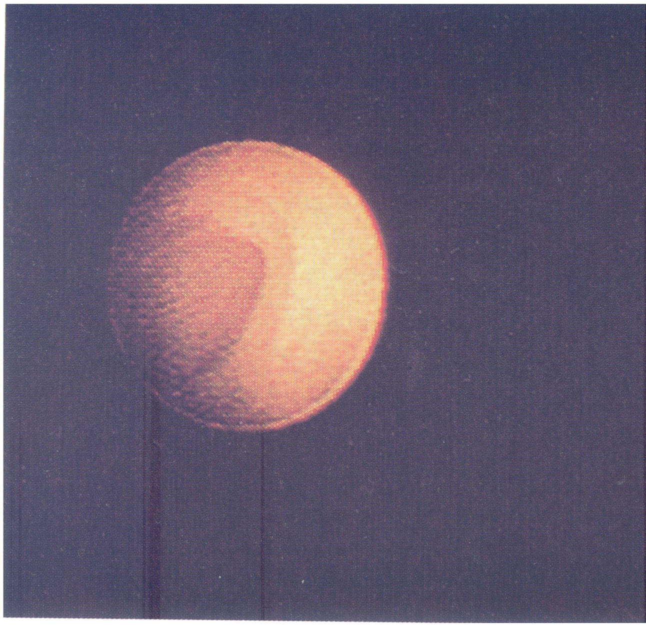

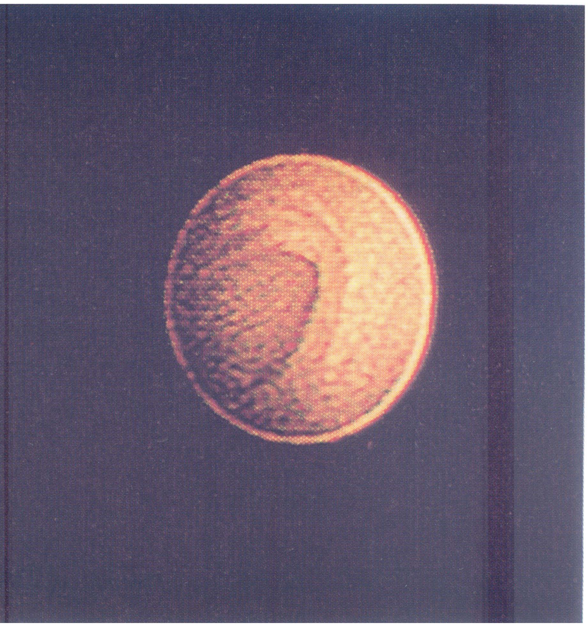

FIGURE 7A Diagnosis of chronic pancreatitis by ERCP. Stenosis with scar is endoscopically observed inside the lumen of the main pancreatic duct.

FIGURE 7B The enhanced image obtained in Peak 2 adaptive enhancement. Fine structure of the pancreatic mucosal pattern is well enhanced.

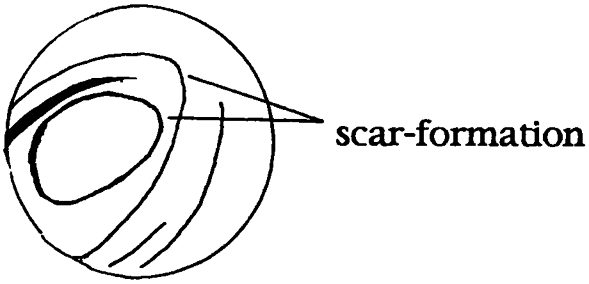

FIGURE 7C Scheme of the endoscopic picture.

TABLE I Studies on the mucosal patterns of pancreatic cancer and chronic pancreatitis, using image processing

| | Peak2 | Peak3 | Peak5 |
|---|---|---|---|
| Clarification of Sharpness of construction of lesions (n=14) | 14(100%) | 8(57%) | 0(0%) |
| Image color (n=10) | 8(80%) | 5(50%) | 0(0%) |
| Poor image quality (n=14) | 0(0%) | 9(64%) | 14(100%) |

in 1989[1,2]. Our fiberscope, with a 0.8mm external diameter, has been used routinely following endoscopic retrograde cholangiopancreatography (ERCP) at an outpatient clinic. This is because it is easy to insert into the normal papilla of Vater without endoscopic sphincterotomy.

This procedure makes it easier to clinico-histologically detect small lesions of the duct in malignant and chronic pancreatitis. We have stressed that it is very useful for the differentiation between local stenosis or elevated lesions of the main pancreatic duct in pancreatic cancer and chronic pancreatitis.

We devised to improve the image quality and visual potential of the ultra-thin pancreatoscope using a sequential video converter with a high-grade charge-coupled device[2]. This could make it easy to obtain a favorable image condition due to the sequential electronic scope system with excellent color reproducibility, and process images by a computer.

Using the IP system, we evaluated the sharpness of the construction of the lesions, peripheral color and image quality in pancreatic cancer and chronic pancreatitis. We specifically used a 14-inch TV monitor, because it is hard to clearly detect images on a 35-mm slide[5]. Additionally, we studied real-time image processing on a TV monitor. The most favorable images were obtained in Peak 2 of adaptive enhancement, having an excellent effect to clearly detect the characteristic mucosal patterns in pancreatic cancer and chronic pancreatitis.

A new electronic endoscope containing real-time adaptive enhancement has been developed. We are convinced that this method would be very useful in improving the diagnostic ability of pancreatic cancer.

### References

[1] Tajiri, H., Yoshino, M., Yoshida, S. *et al.* Clinical application of an ultra-thin cholangiopancreatoscope [in Japanese]. *Gastroenterol Endosc.* 1989; **31:** 1247–1251.

[2] Tajiri, H., Kobayashi, M., Niwa, H. *et al.* Clinical application of an ultra-thin pancreatoscope using a sequential video converter. *Gastrointest Endosc.* 1993; **39:** 371–374.

[3] Kobayashi, M., Tajiri, H., Fukushima, Y. *et al.* The development of an ultra-thin pancreatoscope applying the sequential video converter [in Japanese]. *Gastroenterol Endosc.* 1991; **33:** 1385–1390.

[4] Honda, T., Takeshita, K., Habu, H. *et al.* Observation of the gastric mucosal microstructure using magnifying electronic endoscopy and an image processing of band enhancement [in Japanese]. *Gastroenterol Endosc.* 1992; **34:** 792–799.

[5] Yamagata, S., Ohida, M., Arakawa, T. *et al.* Diagnosis of the extent of superficial spread of gastric cancer by spacial image processing [in Japanese]. *Progress of Digestive Endoscopy.* 1993; **42:** 77–80.

